# Supplementary material for: Constitutive expression of Cas9 and rapamycin-inducible Cre recombinase facilitates conditional genome editing in Plasmodium berghei
Source: Sci Rep. 2025 Jan 23;15:2949. doi: 10.1038/s41598-025-87114-4 (PMC11758014; doi:10.1038/s41598-025-87114-4)
Supplement: Supplementary file 1 — Supplementary Material 1 [file 41598_2025_87114_MOESM1_ESM.pdf]

Supplementary information

Constitutive expression of Cas9 and rapamycin-inducible Cre recombinase facilitates conditional genome editing in *Plasmodium berghei*

Samhita Das[1], Tanaya Unhale[1], Carine Marinach[1], Belsy del Carmen Valeriano Alegria[1#], Camille Roux[1], Hélène Madry[2], Badreddine Mohand Oumoussa[2], Rogerio Amino[3], Shiroh Iwanaga[4], Sylvie Briquet[1¶*], Olivier Silvie[1¶*]

[1]Sorbonne Université, Inserm, CNRS, Centre d'Immunologie et des Maladies Infectieuses, Cimi-Paris, Paris, France
[2]Sorbonne Université, Inserm, UMS Production et Analyse des données en Sciences de la vie et en Santé, PASS, Plateforme Post-génomique de la Pitié-Salpêtrière, P3S, Paris, France
[3]Institut Pasteur, Université Paris Cité, Malaria Infection and Immunity Unit, Paris, France
[4]Research Center for Infectious Disease Control, Department of Molecular Protozoology, Suita, Osaka, Japan

*Corresponding authors
Email: sylvie.briquet@inserm.fr, olivier.silvie@inserm.fr

[¶]These authors contributed equally to the work

Contents

- Supplementary Figures S1 to S4
- Supplementary Tables S1 to S5

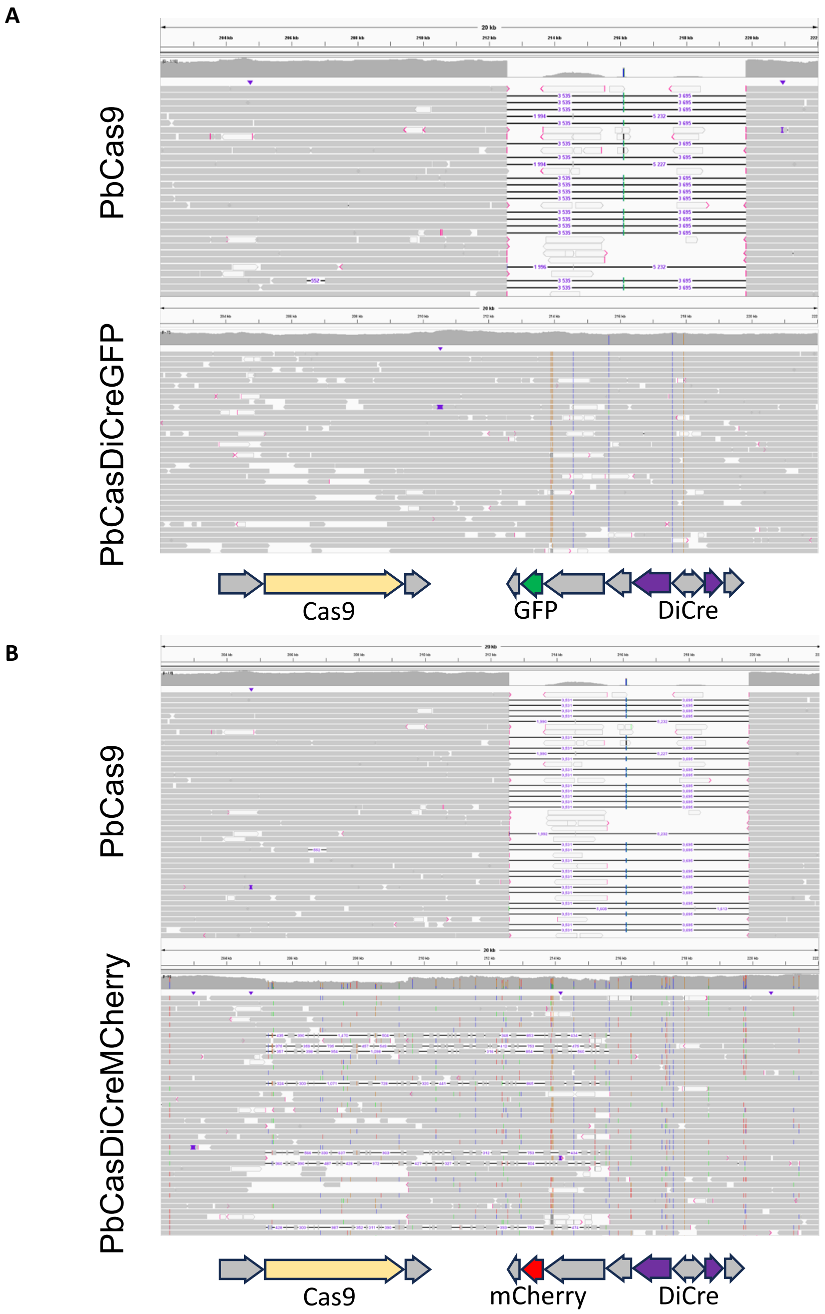

Supplementary Figure S1. **Nanopore sequencing of PbCas9, PbCas-DiCre-GFP and PbCas-DiCre-mCherry parasite lines**

Genomic DNA from PbCas9, PbCasDiCre-GFP and PbCasDiCre-mCherry parasites was sequenced by Oxford Nanopore Technology. Reads were aligned to the expected recombined genome sequences of PbCasDiCre-GFP (**A**) or PbCasDiCre-mCherry (**B**) parasites. Only the *p230p* locus on chromosome 3 is shown. The sequencing coverage was lower with the CasDiCre-mCherry parasite DNA (<50X) as compared to PbCas9 and CasDiCre-GFP samples (70-90X). Low coverage of the HSP70 promoter (upstream of mCherry) and eEF1α bidirectional promoter (driving expression of the DiCre elements) is visible with PbCas9 DNA, likely due to reads originating from the endogenous HSP70 and eEF1α loci on chromosomes 7 and 11, respectively.

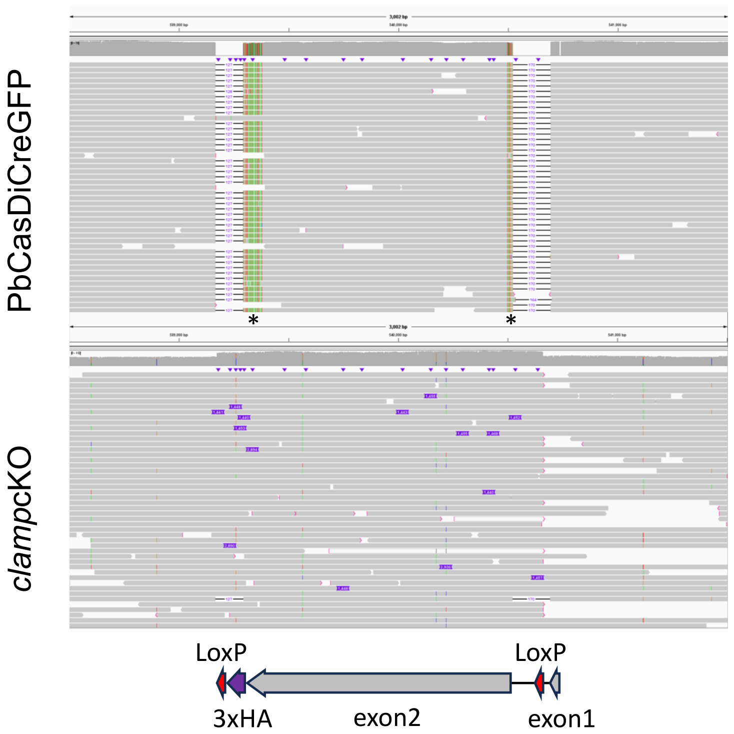

Supplementary Figure S2. Nanopore sequencing of PbCas-DiCre-GFP and *clamp*cKO-HA parasite lines

Genomic DNA from PbCasDiCre-GFP and *clamp*cKO-HA parasites was sequenced by Oxford Nanopore Technology. Reads were aligned to the expected recombined genome sequence. Only the *clamp* locus on chromosome 5 is shown. Shield mutations introduced in the *clamp* donor DNA at the sgRNA target sites are indicated by asterisks.

A

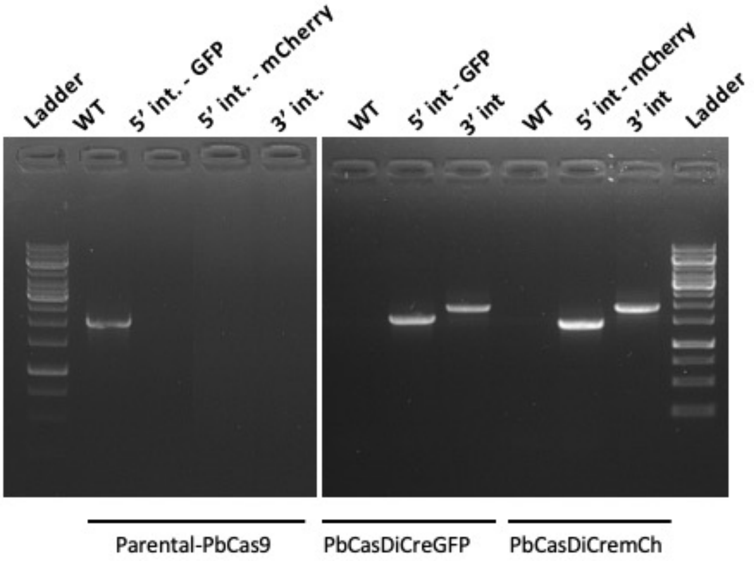

B

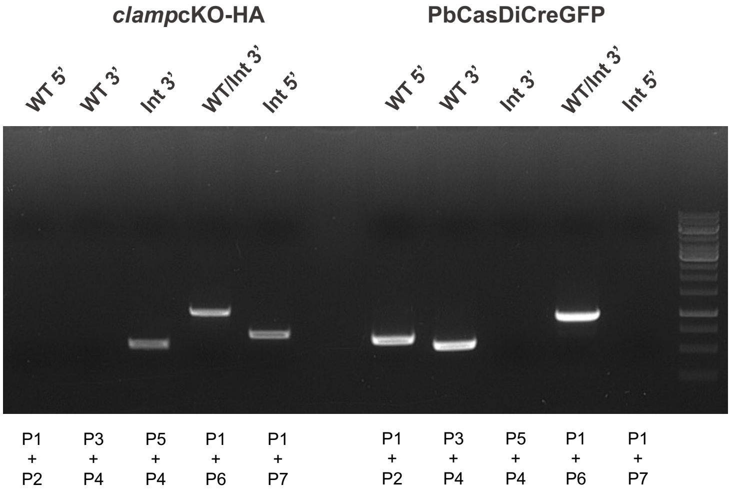

Supplementary Figure S3. Uncropped gel images

The uncropped gel images in A and B correspond to Figures 1C and 3D, respectively.

A

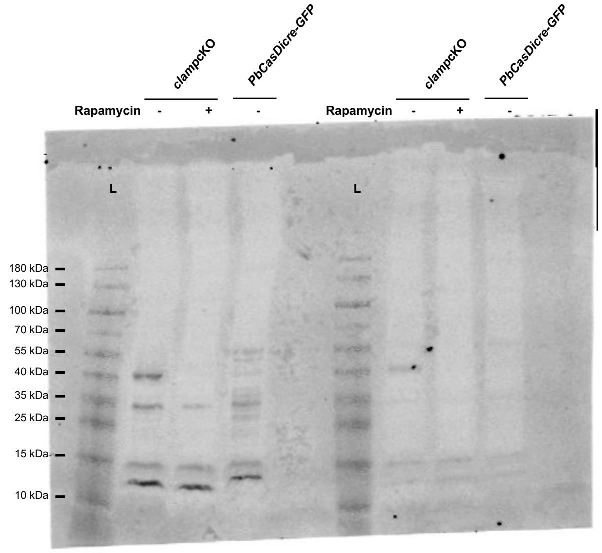

Blot: anti-HA

B

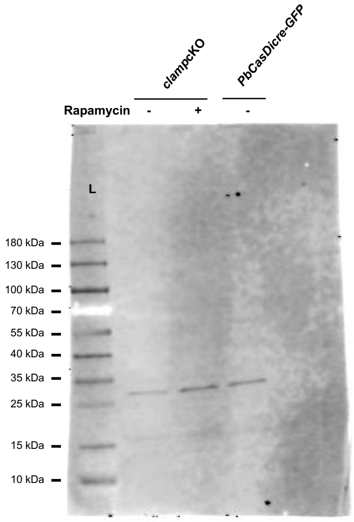

Blot: anti-GFP

Supplementary Figure S4. Uncropped blot images

The uncropped blot images correspond to Figure 4C.

Supplementary Table S1. Sequences of oligonucleotides and synthetic genes used to assemble the chimeric constructs.

	**Oligonucleotide**	**Sequence 5´ → 3´**
**sgRNA oligonucleotides**	p230p sgRNA1for	TTATACAATATTATTGATACTTTTTTTGTTCGAAGCGTTTTAGAGCTAGAA
	p230p sgRNA1rev	TTCTAGCTCTAAAACGCTTCGAACAAAAAAAGTATCAATAATATTGTATAA
	clamp sgRNA1for	TTATACAATATTATTGCGAGCTATGCCTGATCAAAGGTTTTAGAGCTAGAA
	clamp sgRNA1rev	TTCTAGCTCTAAAACCTTTGATCAGGCATAGCTCGCAATAATATTGTATAA
	clamp sgRNA2for	TAAGTATATAATATTGAGCATTTTTTGACATAGGGGGTTTTAGAGCTAGAA
	clamp sgRNA2rev	TTCTAGCTCTAAAACCCCCTATGTCAAAAAATGCTCAATATTATATACTTA
**DiCre repair constructs**	DiCre cassette For	CCCCCTCGAGGTCGACAGTATAAATAGATTCCCTAATGTTT
	DiCre cassette For	CGGGCTGCAGGAATTCGGAACTTAATAAAAAAGAGGAGT
	p230p HR5for	GGGAACAAAAGCTGGGTACCTGAATCAAAAGGAATTATAGGTACTGCATC
	p230p HR5rev	TACTGTCGACCTCGAGTATCGTCATTATATTTTTCTTGATCAATGG
	p230p HR3for	AGTTCTAGAGCGGCCGCATGAAGTTACTATAAATGCATCAGA
	p230p HR3rev	AATTGGAGCTCCACCGCGGGAACATTAATTTTAACAATTGCTGT
	GFP/mCherry cassette for	TACTGTCGACCTCGAGACAGTGTATATTCCCTCAGTTTTCAAATGG
	GFP/mCherry cassette rev	AATGACGATACTCGAGTCAAGTCCAACTATTTATGAATCATTGAAGAG
**clampcKO-HA repair construct**	Synthetic gene	ATGTACATATTTGTGTTTAAAATAAATTAACTCATTATAAAATAATTTTTTTTT<br>CATATTTATTTAAATAATTTTATTTATAAATAAAAAAAATAAAAAAAACTTTAC<br>AATGCCCCCTCCCCAATATATCTATATATTCTTGTAAAAGGTGCGAATTCAATA<br>CATATATATATATATATATATATATGTGTATATTTATTTATCCATTTTTTTAAGGA<br>AAAAAATAACAAAAAAAATAATGGAAAATAAAACAACCAAAAAGCGATCTAAAT<br>ATATATTACTTTAAAAAGACAGGAATGGGAAATGTAGAAAATGTTGCTTTTTTA<br>AATACTGAATTAAAAGTATCAATATATATATATTTTTTTTATATTTGTATATTTAG<br>GGAGAAAAAATTAATTTTCGAATATTTTTCCTAACTAAAATATTATTGCGCAAT<br>AAAACATATGGATAGTGTCGCATAAATGTTTTACACATTTTTATATTTGCCTGT<br>ATTTTATGGAAATAGTTTATAAAAAATTTATTTTGACATAGATGGTACCAAATA<br>GGCACAATGCCAAAAGTCAAAAAAGGAAAGGCAATTAGTATGTTATATTTATGC<br>ATGCCATACTAACA**ATAACTTCGTATAGCATACATTATACGAAGTTAT**TTTGTT<br>GTTTCCATATGAATGCATCATTTACAATAATATATTTTACATATAAATATTATG<br>AAATTGTATTAATGTAAATTCTTTCCTTCAAAAATACCATGTTAGCCCCACCAA<br>TGTCCAAGAACGCGACTCGGGTTTTGAATTTAATAATGGCTATAGGGTTTATAT<br>GCGCAATACCTCAAATTTTAACAACCGTTATGACAAGTTGGAGAGAAGGGGAAC<br>CTATTGAATATAAATATATGTTTAGAGGAAGTGAACATTCTTTATATGTTGATT<br>ACACATATTATGGATTATACAAAGTAATATATGATGATAGACATGTTGAAACAT<br>GGACACAACGAGTTCAAAATATGAGAAATAAAGGTAGTGAAGGAATACAAGTAG<br>GAAGAAATAGCGAATCGAGTGGATCGTTATCTTTATGGACATCTGCATGTCAAG<br>AACCATGTAGGGATTCAATAATAAAACGTATAGAAGCATATGAACGAGTTTCAT<br>TTATTTCATTAGTTTTGTTATGTGGTATTGTTTTAGCATGCTCTCTTGTTGTTA<br>TGTGTGTAGGGTGGAATATATTATTTACAAAAAACATTTTAATATCAATGGGGT<br>GTTTTATGTTATCTTTTATAATTAATGGTGGAATAGGAACATATTGGTATTATG<br>AAACAGATTTATCATGGAATTTAGTAACAAAATCTCAACAATATCCATTTCCAA<br>AATGTTCATATAGTTTTTATATTTTTATGATAACAACCTGTATATATGGTTTAT<br>GTTTTATTGGTTTATTATTGTTAGATTTATCAAATAAAAATTCACAAAAAAAAT<br>CATATATTAATCATGGAAATACCAGAAATATGATATTTGAGGAAGGAAAGAATA<br>TGGCTTATCAACCATTATATGACCATCAAAATAGTATGATGATGCCAAGGAGTG<br>CCAGTTATTCAAATATGATGCATTTTGGAAAGGGAATGGATAATTATCCTTCTA<br>GTTTTATTCCATATAACAATAGTAAAATGAATATGAACAACCCTATGGGAATTA<br>ATAATTCATTAAATATGATGGGGAATAATGTCGGAGGTTATCCAGGATTTAGAA<br>ATATGCCATCAGGAATTGGAAATGGAAATATGATGATGCCTCATCAAAACATGA<br>ATCCAATGTTTTCACCACAAATGTCAAGACAATTTTCATATTCAGGATCGCATT<br>CAATGGGTAATTTTGGAATGATAAATTTCCAAAATAGAAATATTCAAGATATGC<br>GCGCGATGCCCGACCAGAGGGGCTACTCTCGAATGAGCTTCGACGTGAACAACC

		CGTACGGGTCCCAGAAGCAGCATATGTTCTACCCATATGACGTTCCAGACTACG<br>CGTATCCGTACGACGTTCCGGATTACGCTTACCCTTACGACGTACCTGACTACG<br>CTTAAGGATCC**ATAACTTCGTATAGCATACATTATACGAAGTTAT**CCATGGTCT<br>AAAACAGTCAAATAATAATAGTAGGGTTGTTCTATTTATCATAACTATAAAAAT<br>AATCTCATATTTTTGTTATCAAATTTTTTGGAAATTTTCAAAAAAATATATTAT<br>CTTCATATAGGAAATCTTTAGATTGCGCAGGAAAAACTAGTACTCAATTCTTTT<br>CTTAAACTAGCCAAAATAAATAAGATTATGTTTGGCAACATGCACATTCCCATG<br>CTAGGTTCATGTACATGGACATATGCATATACGAGTGTATGCAGATACGAGTTT<br>AGGCCATAAAAAGTGAGAGAATAGTGATAAAAATAAACCTCACAAATTAAAAAG<br>TTATAAATATTTTATCTTAAATTGTAAGTGTTGAATATTTAAAAAAAATGGCAA<br>TTAATATCTGCATATTATTTATTTTTATGTTTGTTTTACTCTTGTATAATTTTT<br>TTTTCATTTTTGAACAATGAATTAACAAAATATTTTCAAATTAAATAATCTTTA<br>ATTTATCCAAGGTATATTATTTTTTTTTTTTTCATTTTC
**Cas-Dicre genotyping**	T1	GTGATAAAATGTCCACCAAAAAAAAGTGG
	T2	CAAAGCACTTACAAGTAAGATGATC
	R1	GATGGAAGCGTTCAACTAGCAGACC
	R2	AACAGTACGAACGCGCCGAGG
	R3	GAGAGCCAAACTGACTATATCTC
**clampcKO-HA genotyping**	P1	CAAACATCGTTAAAAAATATTTTGGTATAATTTTG
	P2	ATTTTTTGACATAGGGGGGGTAATTGCC
	P3	GAGTTTTGATGTAAATAATCCATATGGTTC
	P4	AGAAAAAAAACGATGTATAGTTGGTCTAC
	P5	ACGACGTACCTGACTACGCTTAAGG
	P6	CAGATGTCCATAAAGATAACGATCC
	P7	ATTGTAAATGATGCATTCATATGGAAACAAC

Supplementary Table S2. Quantitative source data corresponding to Figure 2 A-E.

**Fig 2A**	**SG spz numbers/mosquito**	PbGFP	PbDiCre	PbCas-DiCre-GFP	PbCas-DiCre-mCherry		One-way ANOVA
	Exp 1	21590		17272	11381		ns
	Exp 2	5667		3000	5700		P = 0.3897
	Exp 3	13815	3280	6666	3448		
	Exp 4	6041	4742	7714	2741		
**Fig 2B**	**% Dextran-positive cells**	PbGFP	PbDiCre	PbCas-DiCre-GFP	PbCas-DiCre-mCherry		One-way ANOVA
	Exp 1	39,19		34,64	54,42		ns
	Exp 2	30,08		30,65	41,06		P = 0.6032
	Exp 3	34,26	30,07	38,25	30,07		
	Exp 4	58,16	49,99	34,28	52,91		
**Fig 2C**	**% infected cells**	PbGFP	PbDiCre	PbCas-DiCre-GFP	PbCas-DiCre-mCherry		One-way ANOVA
	Exp 1	4,94		1,82	5,88		ns
	Exp 2	3,65		2,33	3,07		P= 0.3150
	Exp 3	3,25	3,18	2,96	2,04		
	Exp 4	4,64	2,01	2,73	1,86		
**Fig 2D**	**UIS4-positive EEFs 24h pi**	PbGFP	PbDiCre	PbCas-DiCre-GFP	PbCas-DiCre-mCherry		One-way ANOVA
	Exp 1	277		196	261		ns
	Exp 2	212		139	120		P= 0.3330
	Exp 3	225	231	215	239		
	Exp 4	251	136	176	192		
**Fig 2E**	**UIS4-positive EEFs 48h pi**	PbGFP	PbDiCre	PbCas-DiCre-GFP	PbCas-DiCre-mCherry		One-way ANOVA
	Exp 1	232		160	253		ns
	Exp 2	120		114	104		P= 0.6529
	Exp 3	236	227	227	233		
	Exp 4	267	144	136	185		

Supplementary Table S3. Quantitative source data corresponding to Figure 2G.

Parasitaemia	PbGFP			PbCas-DiCre-GFP			PbCas-DiCre-mCherry		
	mouse #1	mouse #2	mouse #3	mouse #1	mouse #2	mouse #3	mouse #1	mouse #2	mouse #3
Day 1	0	0	0	0	0	0	0	0	0
Day 2	0	0	0	0	0	0	0	0	0
Day 3	0	0	0	0	0	0	0	0	0
Day 4	0	0	0	0	0	0	0	0	0
Day 5	0,03	0,01	0,12	0,28	0,19	0,21	0,15	0,09	0,08
Day 6	0,77	0,67	0,8	1,5	0,98	1,31	0,47	0,51	0,42
Day 7	4,89	3,83	4,99	3,33	3,41	4,28	3,18	3,89	3,15

Mean parasitemia	PbGFP	PbCas-DiCre-GFP	PbCas-DiCre-GFP	Two-way ANOVA
Day 1	0,000	0,000	0,000	Column factor: ns
Day 2	0,000	0,000	0,000	P=0.0705
Day 3	0,000	0,000	0,000	
Day 4	0,053	0,000	0,000	
Day 5	0,053	0,227	0,107	
Day 6	0,747	1,263	0,467	
Day 7	4,570	3,673	3,407	

Supplementary Table S4. Quantitative source data corresponding to Figure 4A.

Parasitemia	clampcKO-HA untreated					clampcKO-HA rapamycin				
	mouse #1	mouse #2	mouse #3	mouse #4	mouse #5	mouse #1	mouse #2	mouse #3	mouse #4	mouse #5
Day 1	2,45	2,04	1,87	1,54	2,17	2,13	2,14	2,43	2,29	3,16
Day 2	5,58	10,94	7,63	6,22	3,4	0,24	0,39	0,91	0,89	0,98
Day 3	5,77	12,52	8,71	6,96	4,37	0,11	0,11	0,05	0,16	0,18

Mean parasitemia	Untreated	Rapamycin	Unpaired t test	
Day 1	2,014	2,430	ns	P=0.0631
Day 2	6,754	0,682	***	P=0.0007
Day 3	7,666	0,122	***	P=0.0003

Supplementary Table S5. Quantitative source data corresponding to Figure 4C.

Parasitemia	clampcKO-HA untreated			clampcKO-HA rapamycin		
	mouse #1	mouse #2	mouse #3	mouse #1	mouse #2	mouse #3
3h p.i.	0,10	0,09	0,01	0	0	0
24h p.i.	0,06	0,06	0,03	0	0	0
45h p.i.	0,71	0,60	0,18	0	0	0
52h p.i.	1,81	1,78	0,67	0	0	0

Mean parasitemia	Untreated	Rapamycin	Unpaired t test	
3h p.i.	0,067	0,000	*	P=0.0397
24h p.i.	0,050	0,000	**	P=0,0037
45h p.i.	0,497	0,000	*	P=0,0185
52h p.i.	1,420	0,000	**	P=0,0097
